# Vaginal Microbiota and HPV in Latin America: A Narrative Review

**DOI:** 10.3390/microorganisms12030619

**Published:** 2024-03-20

**Authors:** Eduardo Tosado-Rodríguez, Ian Alvarado-Vélez, Josefina Romaguera, Filipa Godoy-Vitorino

**Affiliations:** 1 Department of Microbiology and Medical Zoology, Medical Sciences Campus, University of Puerto Rico School of Medicine, San Juan 00935, Puerto Rico; 2 Department of Obstetrics and Gynecology, Medical Sciences Campus, University of Puerto Rico School of Medicine, San Juan 00935, Puerto Rico

**Keywords:** microbiota, vagina, HPV, cancer, dysbiosis

## Abstract

With the expansion of human microbiome studies in the last 15 years, we have realized the immense implications of microbes in human health. The human holobiont is now accepted, given the commensal relationships with bacteria, fungi, parasites, viruses, and human cells. The cervicovaginal microbiota is a specific case within the human microbiome where diversity is lower to maintain a chemical barrier of protection against infections. This narrative review focuses on the vaginal microbiome. It summarizes key findings on how native bacteria protect women from disease or predispose them to damaging inflammatory processes with an emphasis on the role of HPV infections in Latin America, one of the world’s regions with the highest cervical cancer prevalence.

## 1. The Vaginal Biota, Its Community State Types (CSTs) and Ethnical Considerations

The human microbiome comprises Eukarya, Bacteria, Archaea, and viruses and can be found in all body niches. Animals are not merely individuals composed of eukaryotic cells but a complex community of symbionts interacting directly with their hosts (host eukaryotes holding a diversity of microbial eukaryotes and prokaryotes). This is coined as the holobiont, a term first described in the context of the coral and their symbiotic bacteria and is now applied to all living systems [1]. These microbial guests offer key extra metabolic pathways affecting vital physiological functions. Human symbionts and their genetic and genomic composites are considered the “human microbiome”. These symbiotic communities differ significantly across different body niches, and within individuals, the vaginal microbiota is a unique case of a relatively simple community [2]. The human vagina is colonized by diverse microorganisms that make up the normal microbiota that responds to the selective pressure of glycogen [3]. Based on species’ dominance and to facilitate the characterization of the microbial communities, the vaginal microbiota is estimated to have five Community State-Types (CST) per the characterization of 16S rRNA genes [4]. Each microbial community represents different groups of dominant Lactobacilli [5]. *L. crispatus* is the most abundant species in CST-I, followed by *L. gasseri*, *L. iners*, and *L. jensenii* in CST-II, CST-III, and CST-V, respectively [6]. On the contrary, CST-IV includes fewer *Lactobacillus* species and more anaerobic bacteria, including *Prevotella*, *Atopobium*, *Sneathia*, and *Gardnerella*, which have been linked to bacterial vaginosis [7]. A distinction between CST IV-A and CST IV-B is that the latter contains some BV-associated bacteria (BVAB), while CST IV-A is often associated with low Nugent scores. *Atopobium vaginae* are frequently found in moderate quantities in both CST IV-A and IV-B—the relative abundance of *Lactobacillus* spp., *G. vaginalis*, *A. vaginae*, and Ca. *L. vaginae* might be low in CST IV-C samples, characterized by a wide array of facultative and strictly anaerobic bacteria [2]. As a result, CST IV-C was divided into five sub-CSTs: CST IV-C0 is a balanced community with a moderate proportion of *Prevotella*. *Streptococcus* dominates in CST IV-C1, *Enterococcus* in CST IV-C2, *Bifidobacterium* in CST IV-C3, and *Staphylococcus* in CST IV-C4 [2].

The diversity of the vaginal microbiota exhibits considerable regional and ethnic heterogeneity. This is evidenced by the increased bacterial diversity in African and Hispanic women and the higher prevalence of *Lactobacillus* species in Caucasian and Asian populations [8]. In contrast to African-American and Hispanic women, who mainly exhibit a CST-IV profile, Caucasians often have a CST-I-dominant microbiota [9]. Several studies have explored the role and impact of the predominance of *L. iners* over the healthy and diseased cervicovaginal state. These studies have concluded that further research is required, particularly implementing deep sequencing approaches differentiating between strains and clones of *L. iners*, to understand the community dynamics and its effects on vaginal health [10]. It is important to state that the mere presence or dominance of *L. iners* does not represent disease, as studies have described.

*L. iners* are commonly seen in people with cervical intraepithelial neoplasia (CIN) [11], which is consistent with the statement mentioned above of its presence in both healthy asymptomatic conditions as well as vaginal dysbiosis. *L. iners* differs from other *Lactobacillus* species in having a relatively small genome, complicated nutritional requirements (needs supplements, such as sheep or human blood, and an anaerobic environment), and a polymorphic shape, which may indicate a symbiotic or parasitic nature [12]. *L. iners* forms an inerolysin encoded by the genome of *L. iners* strains, a pore-forming toxin like the vaginolysin produced by *Gardnerella vaginalis*. A possible explanation for its complex role in cervical health might be its clonal variations, which, in some situations, support health and, in other cases, are linked to dysbiosis and a propensity for illness [12]. Altogether, alterations in cervical bacterial populations appear to trigger immunological dysregulation, supporting a tumor-promoting cervical milieu, therefore having a crucial role during cancer progression.

A diverse cervicovaginal microbiota distinguishes Hispanic women living in Puerto Rico with lower proportions of overall Lactobacilli (35.4%) and dominance of *L. iners* (39.5%) [13]. Despite being in the Caribbean, women living in Puerto Rico vary from other Afro-Caribbean women from Barbados, where *Prevotella* predominates and non-Lactobacillus-dominated profiles (72%), likely due to a more significant African contribution than in Puerto Rico [14]. These findings are in line with earlier results from Hispanic women living in Puerto Rico [13,15], studies of U.S. Hispanics [6], and even in South America, such as Venezuelans [11].

The prevalence of cervical HPV infection in a population-based sample of women in Puerto Rico has been estimated at 29.4% (95% CI 23.2–36.4%), while the prevalence of HR types of infection in the cervix is estimated at 8.4% (95% CI 5.6–12.6%). The prevalence of LR types has been identified as higher, with an estimate of 17.4% (95% CI 13.0–23.0%). The prevalence of HPV co-infection was 17.1% (95% CI 12.6–22.8%), co-infection particularly with HR types has been reported as 4.2% (95% CI 2.1–8.2%), while with LR types estimated as 6.4% (95% CI 3.9–10.4%) [16]. HPV prevalence in other groups is in Costa Ricans (HR-HPV, 35%) [17], Europeans in the Netherlands (20%) [18], Japanese (20%) [19], and Nigerians (HR-HPV, 40%) [20]. Puerto Rican women without cervical lesions or HPV infections often have a diverse cervicovaginal microbial profile [13,15]. They also have a greater risk of HR-HPV infections [13], which renders them more susceptible to cervicovaginal dysregulation and inflammatory disorders. They have shown the dominance of *L. iners* on asymptomatic participants, indicating that a non-*L. crispatus* dominant community may not always indicate severe dysbiosis [13,21].

Besides bacteria, studies in Hispanic women revealed that a cervical yeast—*Malassezia*—could be associated with high-risk HPV infections [13]. However, these results were limited by a failure to address the host response to these microbial changes. In addition, reported data were based on the collection of cervical swabs during a pelvic examination, which falls short of a clear understanding of the host-microbiome frontier, revealing the necessity for further multi-omic studies using approaches such as metabolic, proteomic, and cytokine profile analyses to reveal more detailed processes of the cervical microenvironment [22,23]. Indeed, a recent study implemented predictive models for the first time, with multi-omics integration techniques such as neural networks and Random Forest supervised learning on both immune and cancer biomarker datasets. This study was on Arizonan women with or without cervical neoplasm. Random Forest regressors trained on microbial and metabolic features indicated a close relationship between the vaginal microbiome, metabolome, and genital inflammation involved in cervical carcinogenesis [24]. Sphingolipids and long-chain unsaturated fatty acids, for instance, were excellent predictors of genital inflammation, whereas the vaginal microbiome was best predicted by vaginal pH and amino acid metabolism [24]. Similar efforts are ongoing in Puerto Rico with Hispanic women (2U54MD007600-36 (8538) National Institute on Minority Health and Health Disparities).

## 2. Factors Shaping Vaginal Microbiota Dynamics

The vaginal microbiota, a dynamic and intricately balanced ecosystem, is influenced by a multitude of factors that collectively contribute to its composition and functionality. Age is a crucial determinant in this complex interplay as it is tightly associated with hormonal changes. Also, hormonal fluctuations, mainly changes in estrogen levels, are pivotal in vaginal microbiota dynamics. During reproductive years, high estrogen levels promote the dominance of Lactobacillus species, creating an acidic environment that helps maintain vaginal health [6]. High estrogen levels during the menstrual cycle promote the growth of Lactobacillus, contributing to a healthy vaginal environment [6]. For example, postmenopausal women often experience a shift in microbial composition due to declining estrogen levels, reducing Lactobacillus abundance and increasing susceptibility to infections [5].

Lifestyle choices, including smoking and drinking, also impact the vaginal microbiota. Research indicates that smoking is associated with alterations in microbial diversity, reducing the prevalence of Lactobacillus and elevating the risk of infections [5]. Similarly, excessive alcohol consumption has been linked to disruptions in the vaginal microbial balance, highlighting the intricate relationship between lifestyle factors and vaginal health [7]. Also, using different menstrual products, such as tampons and cups, introduces another layer of complexity. A study revealed that girls utilizing menstrual cups had a 26% lower likelihood of bacterial vaginosis and a 37% higher probability of maintaining an optimal vaginal microbiome compared to those without cups. While there was no evident reduction in the overall risk of sexually transmitted infections (STIs) with menstrual cup use, adjustments for confounding variables like age and sexual activity revealed a decrease in STIs among cup users [25]. Nevertheless, nonoptimal hygiene practices or prolonged use of these products may create conditions conducive to bacterial overgrowth and infections [26]. Mechanical factors associated with these products, such as localized friction, can further impact the vaginal microenvironment, influencing microbial dynamics [26,27].

Sexual intercourse, a fundamental aspect of reproductive health, plays a role in shaping the vaginal microbiota. The introduction of microorganisms during sexual activity can temporarily disturb the microbial balance, but the vagina typically restores its equilibrium shortly afterward [6]. However, factors such as the frequency of sexual activity, the number of sexual partners, and the use of barrier methods can influence the stability and composition of the vaginal microbiota [5].

Exceptional cases, such as bacterial vaginosis (BV), underscore the vulnerability of the vaginal microbiota. BV is characterized by a dysbiotic shift, marked by decreased Lactobacillus and an overgrowth of other bacteria. Various factors, including sexual activity, douching, and intrauterine device usage, have been implicated in elevating the risk of BV [5]. Overall, the dynamics of the vaginal microbiota are shaped by a multifaceted interplay of age, lifestyle choices, menstrual practices, sexual activity, hormonal levels, and specific conditions such as bacterial vaginosis. Understanding these factors is essential for promoting women’s reproductive health and preventing the onset of infections.

## 3. The Biology of Human Papillomaviruses and Cancer

Papillomaviruses are dsDNA viruses that belong to the Papillomaviridae family, characterized by an icosahedral capsid of 72 capsomers and a size ranging from 50 to 55 nm. These viruses are widely distributed in mammals but are highly species-specific, as with Human Papillomavirus (HPV) infecting only humans. Fourteen HPV species comprise almost 200 genotypes, from which only 40 can infect epithelial cells. These 40 species could be subclassified as cutaneous or mucosal based on their tissue tropism [28]. HPVs are composed of viral proteins described with a nomenclature based on their open reading frames (ORFs), which are expressed from polycistronic mRNAs. These are named the Early Region (E1-E8 ORFs), which codifies for proteins necessary for viral replication, and the Late Region (L1-L2 ORFs), codifyies for viral capsid proteins; HPVs are classified into different types based on the characteristics of the L1 protein.

Certain HPV types have been associated with infecting specific body sites and with diverse clinical presentations. Low oncogenic risk (LR) HPV (6, 11, 40, 42, 43, 44, 54, 61, 70, 72, 81, 89) infections are linked to benign epithelial lesions such as anogenital and oropharyngeal warts. In contrast, high oncogenic risk (HR) HPV genotypes (16, 18, 31, 33, 35, 39, 45, 51, 52, 56, 58, 59, 66, 68) have been classified as such, based on their association with the pathogenesis of invasive tumors and the high risk they represent for the development of Cervical Cancer (CC), primarily squamous cell carcinoma [28]. According to research, HPV HR 16 and 18 are present in around 70% of invasive carcinomas of CC, but when exploring the presence of any HR-HPV regardless of their genotype, this number goes up to 99.8% [29]. In addition, there are HPV types with an “undetermined risk” (3, 7, 10, 27, 28, 29, 30, 32, 34, 55, 57, 62, 67, 69, 71, 74, 75, 77, 83, 84, 85, 86, 87, 90, 91) including those whose oncogenicity has not yet been fully defined [29].

Infection with HPV in sexually active women has a high prevalence (75% of sexually active female patients over 25 years old), but these tend to regress spontaneously during the first two years in 90% of the cases of HR-HPV [30]. During that period between infection and clearance, low-grade cytological abnormalities may be clinically detectable in screening but are usually transient. The infections that are not cleared are defined as “persistent infections”, which are more common in HR-HPV and are an important factor in the pathogenesis of precancerous and cancerous lesions [30]. These persistent infections increase the risk of developing histopathologic high-grade cervical intraepithelial lesion HSIL (CIN2 and CIN3) or invasive CC in the case of the 30–44 age group by a factor of 11, in the 45–54 group by a factor of 35, and in those who are 50 years old or older by a factor of 49 [31]. The HR HPV types most associated with high affinity for neoplastic progression are type 16 (in 50% of cases) and 18 (in 20% of cases). An additional 19% could be attributed to HPV types 31, 33, 45, 52, and 58 [31]. Results from the Pap-Smear could be classified using the Bethesda system as negative for Intraepithelial Lesion or Malignancy (NILM), low-grade squamous intraepithelial lesion (LGSIL, and includes the CIN-1 nomenclature) and high-grade squamous intraepithelial lesion (HGSIL and includes the CIN-2 and CIN-3 nomenclature) [32]. The grade of dysplasia described in these lesions has the potential to regress spontaneously or to progress. LGSIL has a low potential for progression and a high potential for regression [33], while HGSIL (CIN-2 and 3) has a higher potential for progression and a lower potential for regression than LGSIL [34]. Based on this information, LGSIL (CIN-1) management is usually observation, while management of HGSIL is mostly treated with lesion removal. Based on this information, management for LGSIL (CIN-1) is usually observation, while management of HGSSIL is mostly treated with lesion removal.

## 4. The Interplay of the Microbiota, HPV, and the Immune System

Persistent oncogenic infection with Human Papillomavirus (HPV) is the etiological agent necessary for developing cervical cancer, with a prevalence of 90–100% in cervical abnormalities, ranging from Low-grade squamous intraepithelial lesions to severe dysplasia and cancer [33]. Besides persistent HPV infection, other factors influence cervical disease progression, including lifestyle, genetic predisposition, immunosuppression, early onset of sexual activities, and multiple births [34,35]. Nevertheless, the reason why only a fraction of women acquires a long-lasting persistent HR-HPV infection that puts them at risk for developing Cervical Intraepithelial Neoplasia (CIN) remains unknown. One potential explanation for this variation could be the cervicovaginal microbiota and its related processes, as shown in a recent study that suggests an association between certain bacterial community types of the vaginal microbiota, HPV infection, and HPV-related disease, eventually leading up to carcinogenesis [11,15,36]. Also, previous studies showed that changes in processes like bacterial vaginosis, cervical inflammation, and increased vaginal pH affect susceptibility to cervical HPV [37].

Since cancer is a metabolic disease that alters several cellular pathways [38], cytokine profiling can provide insightful information on cervical dysbiosis’s impact on cancer development. Because the vaginal microbiome and the mucosal immune system interact closely, microbial composition changes may impact the body’s local immunological response [39]. Cytokines are one of the central signaling peptides of the immune system, and when dysregulated, they are usually associated with altered body states [40]. Also, inflammation in the cervicovaginal area has been associated with specific microbial taxa and is directly associated with cancer progression and metabolic alterations [23]. Thus, assessing cytokine profiles provides a broader understanding of cervical carcinogenesis and expands our understanding of host-microbe interactions. In addition, there are very few studies using cervical lavages for cytokine profiles associated with cervical cancer, even less in a cohort of Hispanics.

Cytokines are divided according to the physiological impact they cause on the host. There are two main types: TH1 and TH2 type cytokines. Th1 cytokines are immune-stimulating and work as tumor-suppressing cytokines [41]. Therefore, they are crucial for an adequate anti-tumor response. On the contrary, Th2-type cytokines are mainly associated with humoral immunity and immune inhibitory responses [42]. In addition, it is relevant to mention that continuous or dysregulated expression of Th1 cytokines promotes chronic inflammation, eventually causing DNA damage and making cells more susceptible to transformation [41]. Therefore, pro-inflammatory cytokine levels can indicate favorable tumor development environments.

Th1 cells produce type I cytokines (e.g., IL-2, interferon), whereas Th2 cells secrete type II cytokines (e.g., IL-4, IL-5, IL-6, IL-10, and IL-13) [43]. Dysregulated Th1/Th2 levels are critical contributors to the immune response in some diseases and can promote changes in cytokine levels [43]. Therefore, cytokine profiles can reflect the local immune state of the cervix. Many cytokines are markedly altered in cervical precancer and cancer, especially in advanced cancer with metastasis. Excess cytokines, such as IL-6 and IL-2, are caused by persistent high-risk HPV viral infection and can be related to tumor growth [44]. On the contrary, in the initial stages of the disease, some cytokines are associated with inhibiting HPV replication and tumor suppression, including IL-1, TNF-α, TGF-β, and IFN-α [44].

The knowledge of the importance of the microbiota in preventing and treating cancer is growing. In homeostasis, the microbiome stays in a dynamic equilibrium. Still, in the cervix, high bacterial diversity and low levels of lactic acid bacteria can lead to bacterial imbalance and inflammation, called dysbiosis [45]. For instance, lactic acid (a *Lactobacillus* metabolite) induces the release of the anti-inflammatory cytokine IL-10 and lowers the synthesis of the pro-inflammatory cytokine IL-12 in dendritic cells. It also decreases the cytotoxicity of natural killer cells [46]. This demonstrates the significant influence of microbiome metabolites on the immune response.

Additionally, certain *Lactobacillus* can influence cellular and humoral immunity, stimulate T-cell growth and differentiation, and further boost immunological recognition and B-cell growth [47]. Various studies illustrate how *Lactobacillus casei* and *Lactobacillus rhamnosus* may operate as anticancer agents by promoting the development of NK cells and dendritic cells [48,49]. However, there are not yet clear vaginal probiotics with high efficacy. According to Wang et al., an increase in *Lactobacillus* spp. was associated with a decrease in the detection rate of high-risk subtype HPV infection, cervical intraepithelial neoplasia, and cancer [50]. Overall, microbial dysregulation causes the release of several pro-inflammatory cytokines and chemokines, leading to a local inflammatory response and raising the body’s sensitivity to infection and the risk of cancer.

Cervical cancer remains the fourth most prevalent cancer in women globally, with notable disparities observed among Hispanic women compared to Caucasian women [51]. Therefore, it still represents a substantial burden to public health systems. Despite increased HPV vaccination rates, we still see an endless number of CC cases per year, indicating that other factors, such as a cervical microbiome, might play a vital role in this process. The cervicovaginal microbiome has been well-characterized, and specific microbial community changes or taxa changes have been associated with reproductive health [7]. Moreover, studies have demonstrated that changes in CSTs from *Lactobacillus* dominant states to polymicrobial states are one of the main risks for transitioning from persistent high-risk HPV infection to Cervical Intraepithelial Lesions (CIN) [52]. More cross-sectional and longitudinal studies, including high-risk populations in Puerto Rico and in many other Latin-American countries, are imperative so new microbiome-based therapeutic interventions can be developed [12,15].

## 5. Cervical Cancer in Latin America and the Caribbean

According to the World Health Organization (WHO), cervical cancer (CC) is the leading cause of death among women in Latin America and the Caribbean (LAC). Despite the current understanding of the pathophysiology, the relationship between High-Risk Human Papillomavirus infection (HR-HPV), and prevention methods, CC accounts for the death of 35,700 women each year in the Americas, with 80% of these cases being in LAC. In 2018, 56,000 new cervical cancer cases were diagnosed, and 28,000 deaths occurred in LAC and the Caribbean. Incidence and mortality have decreased over the past decade this has not been the case for Puerto Rico [51]. LAC countries are still over the elimination threshold of 4 per 100,000 [53]. Here lies the importance of addressing this topic to understand further the factors that could contribute to the high burden of CC in LAC women and eventually direct political, research, and economic efforts toward eradicating this preventable disease.

Risk factors for the development of CC can be classified as HPV-related and non-HPV-related, which are cofactors for developing neoplasia alongside the HPV-HR infection. Some HPV-related risk factors are early onset of sexual activity, multiple sexual partners or high-risk partners, history of sexually transmitted infections, early age at first birth, immunosuppression, and history of cervical squamous intraepithelial neoplasia (IN) [54]. Other risk factors non-HPV-related are low socioeconomic status, limited access to healthcare and cancer screening programs [55], oral contraceptive use [56], cigarette smoking [57], and possible genetic polymorphism such as those who regulate immunity, susceptibility [58], cytokine production [59], angiogenesis [60], tumor suppressor pathways [61], and activators of transcription pathways. Many studies have explored the relationship between HPV and CC, the natural history of HPV infection, and the evaluation of HPV vaccines, but this has not contributed to a faster decrease in incidence or mortality in LAC [62].

Further understanding of the epidemiologic trends of CC in LAC must be explored through a holistic approach of factors and cofactors particular to the region and women who live in it. In 2022, the United Nations CEPALSTAT portal showed that the population has continuously increased, total fertility has decreased to 1.9, and average life expectancy for women in the LAC region has increased to 75 years [63]. This comes in combination with data from 2021, which shows that 12.9% of the population lives in extreme poverty, and 9.3% are unemployed, which means that 204 million people did not have enough income to support their basic needs [63]. Sustaining development of a growing aging population without sufficient economic resources to obtain basic needs or healthcare leads to a complex scenario where accessible screening and vaccination programs are imperative to prevent increased CC incidence and mortality.

Some of the already mentioned risk factors for the development of CC have been studied in LAC. A study in Colombia showed that HPV infection started at coitarche (first sexual intercourse) and that women aged 15–19 are sexually active, and infection prevalence can be as high as 42.5% [64]. The International Agency for Research on Cancer (IARC) conducted HPV surveys, including in women from Argentina, Chile, Mexico, and Colombia, which showed an increase in HPV positivity for women with two lifetime sexual partners compared to those with only one [65]. The ESTAMPA study in Latin America identified the most frequent HPV-HR genotypes in CIN 3 histology as HPV 16, 52, 58, 33, 18, and 39 [66]. An essential but almost unexplored variable comes into play with sexual partner behavior, in which LAC men tend not to remain monogamous after marriage or cohabiting. At the same time, women do [67]. When we analyze studies focused on oral contraceptives (OCP) usage in LAC, many differences are found based on the diversity of family planning situations between countries. Still, the OCP usage prevalence can range from 40% or less to almost 80% in other countries [68].

Tobacco smoking in women, which has been associated with the development of CC, has been estimated from 2% to 26% in LAC countries, while the worldwide estimate in 2015 was 5.4% [69,70]. A study directed their efforts into understanding the relationship that could exist between income level and smoking prevalence with anal, cervical, and vulvar cancer.

The evaluation was conducted by examining data from the Surveillance, Epidemiology, and End Results 21 (SEER-21) database, median family income data from the 2011–2015 American Community Survey by the Census, and county-level smoking rates from the National Cancer Institute’s database for small area estimates related to cancer. The data were arranged into four groups based on the population distribution among counties and analyzed to calculate changes in incidence rates annually using the National Cancer Institute’s Joinpoint Regression Analysis software. Statistical analysis indicated that between 2000 and 2018, the incidence of HPV-related cancers, as well as the specific types of these cancers, were higher in counties with the lowest household incomes and those with higher rates of smoking [71].

The diversity of countries and cultures, lifestyles, and sexual behaviors in the LAC region creates a complex interrelation of factors and cofactors that cannot be described thoroughly with a brief global summary. To this end, we will highlight the case of three countries and their situation with HPV and CC: Venezuela, Costa Rica, and Puerto Rico.

### 5.1. Venezuela

The National Tumor Registry of the Ministry of Popular Power for Health (MPPS) of Venezuela has registered that CC is the second most common cause of death in women aged 15–76. Because of this, a prospective cohort study was designed to assess the prevalence of HPV infection and resulted in 37.40%, a finding surpassing the worldwide prevalence of 9–13%. The study found that the most common serotypes infecting women in Venezuela are HPV 18 and HPV 16, which differs from previous projects that present HPV 16 as the most common. Coinfection with multiple HR-HPV serotypes was also present in 53.93% of the positive samples. The conclusions of the study can be summarized as (a) the cohort demonstrated a low percentage of infections that remain persistent, (b) a high percentage of regression of cytological and colposcopy lesions, and (c) the incidence rate is very similar to that reported by other LAC countries but are higher in comparison with European countries [72].

To further understand the situation of HPV and CC in Venezuela, risk factors have to be taken into consideration, including the fact that the HPV vaccine has not been introduced as a public health policy (same in Cuba and Nicaragua) [73]. In 2021, the fertility rate of live births per woman was 2.3, the use of OCPs and hormonal contraception prevalence was 21.1%, and cervical cancer screening in women aged 25–64 every three years was 35% [74].

In 2012, in the Bolivar State, one of the 24 federal divisions of Venezuela, the CC incidence and mortality were 28.46 and 12.90 per 10,000 women, respectively, which makes it the most frequent cause of cancer death in this state, per the MPPS. When exploring the region’s characteristics, we stumble upon descriptions such as low-income regions with difficult access by land, complex geographical features, and indigenous cultures, all of which combine to hinder the implementation and development of solid screening and diagnostic programs to detect HPV infection and early precancerous lesions. Some crucial studies have been performed in Venezuela focused on estimating HPV prevalence in the context of urbanization level and vaginal microbiome. Venezuelan populations showed the coexistence of HR and LR-HPV risk genotypes in the same women, which highlights the importance of considering the presence of different genotypes and how they modify the risk of HPV-related diseases [75].

Cervicovaginal microbiome analysis led to the identification of microbial taxa that tend to be overrepresented in mestizos, which included *Mobiluncus mulieris* and *Prevotella* sp., while on urban Amerindians there was a prevalence of *Brevibacterium linens* and *Peptoniphilus lacrimalis*. Microbial diversity was not found to be associated with HPV status as in most other studies already published [21]. As of early 2024, HPV vaccines are neither available nor affordable to most in Venezuela.

### 5.2. Costa Rica

HPV vaccination was first recommended in 2006 [76]. The NIH developed in Costa Rica the CR Vaccine Trial (CVT), a phase III randomized clinical trial, which provided the initial data that one dose of the HPV vaccine could provide durable protection against HPV infection [77]. In Costa Rica, current estimates show that 367 women are diagnosed yearly with CC, and 192 die from this disease [74]. It is currently the fifth most diagnosed cancer among women in Costa Rica. The crude incidence rate of cervical cancer is 14.4 (in Venezuela, 25.7, and in Puerto Rico, 9.2–13) [51,74].

In Costa Rica, HPV infection with HR serotypes 16/18 at any given time was estimated at 3.3%, and 62.9% of the invasive cervical cancers are associated with serotypes [74]. In 2021, the smoking prevalence was 6.3%, the fertility rate of live births per woman was 1.8, the use of OCPs and hormonal contraception prevalence was 22.7%, cervical cancer screening in women aged >35 every two years was 35.4%, and HPV vaccination in 2019 was of 98% for first dose and 39% for last dose in Costa Rica [74]. Studies and HPV vaccine efficacy trials have been conducted in Costa Rica, which have reported protection against CIN 3 and CC development [78]. Indeed, cervical cancer mortality in Costa Rica was the lowest in Central America, including Mexico [74]. Recent studies have shown that HPV vaccination reduces HPV testing positive predictive values, but the reduction in HPV-associated lesions was not observed, which might be explained by lesions associated with non-vaccine HPV types. In addition, vaccines are given at an early age and will still not have an impact on women of older ages [79].

The efforts towards preventing cervical cancer have led to various questions about the factors that interplay in its pathogenesis. One of the most consistent interrogations is why certain HPV genotypes are more related to persistent infection and the development of cervical cancer. Some research teams have explored the role that cervicovaginal microbiome (CVM) has over the natural history of HR-HPV infection. The study to be described was performed in Costa Rica, particularly within the placebo arm of the Costa Rica HPV Vaccine Trial that included women aged 18–25. The project’s methodology consisted of collecting cervical samples from two distinct visits from women with a history of HR-HPV (*N* = 273 women). These samples were analyzed for HPV infection clearance, persistence, progression to cervical intraepithelial neoplasia grade 2 and 3 (CIN2/3), and CVM by amplification and sequencing the bacterial 16S V4 rRNA gene region and the fungal ITS1 region. The results of this study identified that the abundance of *Lactobacillus iners* was related to the clearance of HPV infection, while Gardnerella vaginalis abundance was related to disease progression to CIN2/3. The group also obtained results in terms of Shannon diversity, where Gardnerella vaginalis dominance was associated with a more diverse CVM, which was also significantly associated with progression to CIN2/3. The association between the cervicovaginal microbial community composition and HPV-related disease progression in a longitudinal cohort allows the identification of additional protective or risk factors inherent to a particular population. Characterizing CVM compositions that increase infection clearance or less disease progression opens a gap for future research that strives to develop therapy that promotes protective microbial communities [12].

### 5.3. Puerto Rico

The case of CC rates rising in Puerto Rico is surprising. Being a US territory with many HPV vaccination campaigns makes it a special case within LAC. Hispanics living in the United States (US) have a higher CC incidence and mortality when compared to non-Hispanic whites [80], and Hispanics living in Puerto Rico have even higher incidence rates. The heterogeneity of the Hispanic population living in the US makes it imperative to monitor and assess the determinants of health that affect HPV-related disease risk. These subgroups could be organized by ancestry, country of origin, generational status, demographic characteristics, place of residence, and socioeconomic status [81]. Puerto Rico (PR) has the highest incidence of CC of all the US and its territories, with a value of 11.7 per 100,000, while the US is 7.4 per 100,000 [51]. Some of the characteristics found in PR that could influence this high incidence of CC are higher seroprevalence of HPV-HR types when compared to the US and that only 79.3% of women aged 21–65 had a PAP test in the last three years [81]. It is imperative to develop public health systems in PR to continuously monitor the already mentioned risk factors for the development of CC and facilitate screening programs. A factor that could help reduce the burden of HPV and CC in PR is that 91.7% of the population has healthcare coverage, which facilitates access to HPV vaccination and screening for cervical cancer. Another important aspect is the existence of the Puerto Rico Comprehensive Control Plan from 2015 to 2020, which is a plan based on guidelines that consider the importance of increasing HPV vaccination and screening for the prevention of CC [82].

Studies have been conducted in Puerto Rico assessing the aptitude towards HPV vaccination and concluded that knowledge about HPV and vaccination against it is high, but the vaccine uptake is low [83]. This study recommends that physicians and health professionals have a more active approach toward education and HPV vaccination [83]. Other studies have shown the importance of continuing education about the most recent HPV and CC topics in physicians, mainly focused on cancer screening guidelines and HPV vaccination [84]. Recently, the biggest study on CVM in the region, joining both 16S rRNA amplicon sequencing to describe the cervicovaginal bacteria and high-resolution SPF10-LiPA kit to genotype HPVs, concluded that the sample of Hispanics living in Puerto Rico in colposcopy clinics has a high prevalence of high-risk HPV (HR-HPV, 67.3%). The identified genotypes excluded from the 9vt HPV vaccine and *Lactobacillus iners* dominated the cervical microbiome. The diversity of microorganisms linked to bacterial vaginosis decreased in pregnant women in the second and third trimesters, and menopausal women had higher cervicovaginal pH, higher alpha diversity, and percentage of facultative and purely anaerobic bacteria [15]. Ortiz et al. also pioneered the understanding of the prevalence of anogenital HPV infection in women living in the metropolitan area of Puerto Rico. This study included 564 women as subjects, where they completed face-to-face assisted interviews and performed self-collection of anal and cervical samples. These samples were DNA tested for HPV using MY09/MY11 consensus HPV L1 primers and beta-globin as an internal control for sample amplification. The samples that resulted positive were subjected to HPV genotyping using dot-blot hybridization. The results of the HPV DNA tests determined that the prevalence of cervical, anal, and cervical/anal co-infection was 29.4%, 38.6%, and 17.1%, respectively. These positive samples were typified, and the most common oncogenic HPV types detected from cervical and anal samples were 68 (8% vs. 7%) and 16 (5.5% vs. 5.1%).

When these HPV positivity results were analyzed in the context of sexual behavior, they identified that having three or more sexual partners (OR: 2.3; 95% CI: 1.5–3.5) and having anal intercourse (OR: 1.6; 95% CI: 1.1–2.5) during the last year increased the odds of anogenital HPV infection [51].This project exalts the importance of developing deeper knowledge on how sexual behaviors modify the morbidity that anogenital HPV infections have over Hispanic women. Exploring not only the presence or absence of infection but its causative agents down to the level of genotype in combination with the cervicovaginal microbiome and sociodemographic and behavioral variables will aid in identifying those factors that take part in the pathogenesis of HPV-related anal and cervical diseases. These variables could be explored longitudinally to monitor their trends as HPV vaccination increases in the community [16].

The discussion of the factors that influence the risk of developing higher-grade precancerous lesions or CC, in combination with the presentation of the cases of Venezuela, Costa Rica, and Puerto Rico, exemplifies that the LAC region is highly diverse and should be treated as such to be able to reduce or eradicate CC.

## 6. Conclusions

The vaginal microbiota plays a crucial role in women’s health, influencing a range of physiological processes and disease susceptibilities. Community State Types (CSTs) are a way to categorize the composition of the vaginal microbiota based on the dominant bacterial species present. The understanding of CSTs in Latin American women can provide insights into regional variations and health implications. As discussed, most women in Latin America have a microbiota predominantly dominated by *Lactobacillus iners* and some type of Anaerobic bacteria, including *Mobiluncus mulieris* and *Prevotella* sp. *Gardnerella*, associated with progression to CIN2+, which may be associated with subsequent elevation of microbial diversity, although HPV infections did not seem to directly drive microbial diversity increases. The microbial diversity in the vagina and its relationship with Human Papillomavirus (HPV) infection is not direct, as a diversity of microbes is significantly influenced by inflammation and vaginal pH levels. A lower pH, typically around 3.5 to 4.5, is generally maintained by the dominance of lactic acid-producing bacteria, mainly Lactobacillus species, which help protect against infections by inhibiting the growth of pathogenic organisms. The study of vaginal microbiota and CSTs in Latin American women is an important area of research that holds implications for understanding health disparities, improving clinical outcomes, and tailoring public health interventions. Further research is needed to elucidate the complex interactions between host genetics, environmental factors, and microbial communities in this diverse population. The development of further knowledge and surveillance of the already identified risk factors associated with the pathogenesis of CC in every LAC country will allow direct public health efforts. Implementing emergent technology in areas with difficult access, such as accessible primary self-HPV testing, could allow the screening of the community while minimizing visits. Identifying HR-HPV-positive women during a single visit could facilitate directing the limited resources toward following up on those who could develop precancerous lesions or CC [85]. The LAC scientific community should work together towards the goal of eliminating this preventable disease and its burden on our communities through massive HPV vaccination, accessible and adaptable screening programs to the region’s needs, and effective early treatment.

## Data Availability

No new data were created or analyzed in this study. Data sharing is not applicable to this article.
